# Occurrence, antimicrobial resistance, virulence, and biofilm formation capacity of *Vibrio* spp. and *Aeromonas* spp. isolated from raw seafood marketed in Bangkok, Thailand

**DOI:** 10.14202/vetworld.2022.1887-1895

**Published:** 2022-07-31

**Authors:** Sirijan Santajit, Thida Kong-ngoen, Witawat Tunyong, Pornpan Pumirat, Sumate Ampawong, Nitat Sookrung, Nitaya Indrawattana

**Affiliations:** 1 Department of Medical Technology, School of Allied Health Sciences, Walailak University, Tha Sala, 80160, Thailand; 2 Research Center in Tropical Pathobiology, Walailak University, Tha Sala, 80160, Thailand; 3 Department of Microbiology and Immunology, Faculty of Tropical Medicine, Mahidol University, Bangkok 10400, Thailand; 4 Department of Tropical Pathology, Faculty of Tropical Medicine, Mahidol University, Bangkok 10400, Thailand; 5 Biomedical Research Incubator Unit, Department of Research, Faculty of Medicine Siriraj Hospital, Mahidol University, Bangkok 10700, Thailand; 6 Center of Research Excellence on Therapeutic Proteins and Antibody Engineering, Department of Parasitology, Faculty of Medicine Siriraj Hospital, Mahidol University, Bangkok 10700, Thailand

**Keywords:** *Aeromonas* spp, biofilm formation, drug resistance, foodborne, seafood, vibriosis

## Abstract

**Background and Aim:**

Bacteria of the genera *Vibrio* and *Aeromonas* cause seafood-borne zoonoses, which may have a significant impact on food safety, economy, and public health worldwide. The presence of drug-resistant and biofilm-forming phenotypes in the food chain increases the risk for consumers. This study aimed to investigate the characteristics, virulence, biofilm production, and dissemination of antimicrobial-resistant pathogens isolated from seafood markets in Bangkok, Thailand.

**Materials and Methods:**

A total of 120 retail seafood samples were collected from 10 local markets in Bangkok and peripheral areas. All samples were cultured and the *Vibrio* and *Aeromonas* genera were isolated using selective agar and biochemical tests based on standard protocols (ISO 21872-1: 2017). The antibiotic susceptibility test was conducted using the disk diffusion method. The presence of hemolysis and protease production was also investigated. Polymerase chain reaction (PCR) was used to determine the presence of the *hly*A gene. Furthermore, biofilm formation was characterized by microtiter plate assay and scanning electron microscopy.

**Results:**

The bacterial identification test revealed that 35/57 (61.4%) belonged to the *Vibrio* genus and 22/57 (38.6%) to the *Aeromonas* genus. The Kirby–Bauer test demonstrated that 61.4% of the isolates were resistant to at least one antibiotic and 45.61% had a high multiple antibiotic resistance index (≥0.2). PCR analysis indicated that 75.44% of the bacteria harbored the *hly*A gene. Among them, 63.16% exhibited the hemolysis phenotype and 8.77% showed protease activity. The biofilm formation assay demonstrated that approximately 56.14% of all the isolates had the potential to produce biofilms. The moderate biofilm production was the predominant phenotype.

**Conclusion:**

The results of this study provide evidence of the multiple drug resistance phenotype and biofilm formation capacity of *Vibrio* and *Aeromonas* species contaminating raw seafood. Effective control measures and active surveillance of foodborne zoonoses are crucial for food safety and to decrease the occurrence of diseases associated with seafood consumption.

## Introduction

Members of the *Vibrio* and *Aeromonas* genera are facultative Gram-negative bacilli that commonly cause diseases in marine animals and humans [1, 2]. The World Health Organization considers these as a well-known group of foodborne pathogenic bacteria [3–5]. Infections caused by virulent and drug-resistant strains remain a concern for public health in both developed and developing countries. The main cause of these infections is the ingestion of contaminated water, raw seafood, or person-to-person transmission [6, 7]. Humans contribute to the spread of antimicrobial-resistant bacteria in the environment, increasing their contamination levels in foods such as pork, chicken, beef, and seafood [8]. The Center for Disease Control and Prevention revealed that vibriosis causes 80,000 cases of illness each year in the United States, of which 52,000 are due to the consumption of food containing *Vibrio* pathogens. Most people with a foodborne bacterial infection have watery diarrhea. Possible symptoms may range from self-limiting gastroenteritis to severe life-threatening septicemia and necrotizing fasciitis [9, 10]. Some people may also experience further symptoms, such as nausea, vomiting, stomach cramping, fever, and chills. Symptoms usually start within 1 day after infection and last approximately 3 days [11]. The pathogenic strains of major public health concern are *Vibrio cholerae*, *Vibrio parahaemolyticus*, and *Vibrio vulnificus*, which are mostly associated with human infections [12]. However, other *Vibrio*, such as *Vibrio alginolyticus*, *Vibrio harveyi*, *Vibrio anguillarum*, *Vibrio mimicus*, *Vibrio metschnikovii*, and *Vibrio fluvialis*, which have been identified particularly in marine and estuarine environments, are now regarded as emerging human pathogens [6, 13, 14]. Besides the *Vibrio* genus, *Aeromonas* species have also been reported as causative agents of foodborne diseases. Particularly, *Aeromonas hydrophila*, *Aeromonas veronii*, and *Aeromonas caviae* are associated with several types of human infection, including gastroenteritis, skin and soft tissue infection, septicemia, and respiratory infection in immunocompromised hosts [9, 15–19].

Virulence determinants and antibiotic resistance also play a role in the pathogenesis of the above-mentioned genera. Evidence from previous studies [16, 19] shows that these organisms possess several pathogenic factors, such as adhesins, proteases, hemolysins, aerolysin, and cytotoxic enterotoxins, which are all probably involved in human illnesses. The initial toxin produced is hemolysin, which is encoded by *hly*A and it is one of the most significant virulence factors among *Vibrio* spp., expressing a lysis activity against mammalian red blood cells. This pathogenic phenotype is associated with either wound infection or gastrointestinal infection caused by different *Vibrio* species [16, 19]. *V. cholerae*, *V. parahaemolyticus*, and *V. mimicus* are known causative agents of gastrointestinal infection, whereas *V. alginolyticus* and *V. vulnificus* cause wound infection [20, 21].

Moreover, the outbreak of other foodborne organisms is related to biofilms. Bacterial biofilms provide benefits to bacteria by allowing them to survive more successfully in the environment and thus ensuring their transmission through increased survival. The biofilm-producing ability of isolates further complicates the antibiotic resistance issue. The persister cells are enclosed in an exopolysaccharide matrix component adhered to a solid surface, which confers protection from host defense, disinfectants, and also antibiotics [22, 23]. The susceptibility to antibiotics and environmental changes is significantly reduced in biofilm matrixes compared with planktonic cells. Consequently, high concentrations of antimicrobial agents cannot eliminate infectious biofilms, which make their removal from food processing facilities a considerable challenge [24, 25].

Thus, the present study aimed to investigate the occurrence, antibiotic resistance, virulence, and biofilm formation ability of seafood-borne bacteria isolated from retail markets in Bangkok metropolitan area, Thailand.

## Materials and Methods

### Ethical approval

Ethical approval was not required for this study as samples were collected from the market.

### Study period and location

The study was conducted from January 2021 to March 2021. The samples were collected from 10 retail markets in the central and peripheral of Bangkok metropolitan area.

### Seafood collection, pre-enrichment, bacterial isolation, and identification

A total of 120 raw seafood samples of five different food types (fish, n = 35; shellfish, n = 25; shrimp, n = 20; squid, n = 20; and crab, n = 20). All the samples were selected from markets by convenience sampling; they were subsequently stored in sterile bags on ice and were transferred to the laboratory within 2 h. Sample processing was conducted according to the ISO 21872-1: 2017 (Microbiology of the food chain – Horizontal Method for the determination of *Vibrio* spp.) protocol for the determination of *Vibrio* spp., with some modifications [26]. Briefly, 25 g of individual samples was pre-enriched in alkaline peptone water (Merck, Germany) at 37°C for 6 h. Then, the first enriched cultures were further inoculated into alkaline saline peptone water and were incubated at 37°C for 18 h. Subsequently, the overnight cultures were spread onto selective agar plates, thiosulfate citrate bile salts sucrose, and CHROMagar™ *Vibrio* (CHROMagar, Paris, France) for *Vibrio* spp., also the selective media for *Aeromonas* species (HiMedia, India) and the plates were incubated at 37°C for 18–24 h. The suspected colonies were subjected to conventional biochemical assays for the *Vibrio* and *Aeromonas* genera.

### Antimicrobial susceptibility testing

The antimicrobial resistance patterns of the single colonies of the confirmed *Vibrio* and *Aeromonas* species isolated from seafood samples were determined using the Kirby–Bauer disk diffusion method [27, 28] and the selection of drugs was based on their frequent usage in clinical practices and on the guidelines of the Clinical Laboratory Standards Institute (CLSI). Bacterial isolates were tested against a panel of 15 types of antimicrobial agents (Oxoid, UK) belonging to nine classes, namely, ampicillin (AMP, 10 μg), amoxicillin-clavulanate (AMC, 20/10 μg), ampicillin-sulbactam (SAM, 10/10 μg), piperacillin-tazobactam (TZP, 100/10 μg), cefepime (FEP, 30 μg), cefotaxime (CTX, 30 μg), ceftazidime (CAZ, 30 μg), gentamicin (GN, 10 μg), amikacin (AK, 30 μg), meropenem (MEM, 10 μg), imipenem (IMP, 10 μg), chloramphenicol (C, 30 μg), tetracycline (TE, 30 μg), ciprofloxacin (CIP, 5 μg), and trimethoprim-sulfamethoxazole (SXT, 1.25/23.75 μg). The culture plates were incubated at 37°C for 24 h. The inhibition zone diameters were measured and interpreted based on the CLSI criteria (CLSI, 2011). Moreover, the multiple antibiotic resistance (MAR) index was calculated as the ratio between the number of drugs that bacteria were resistant to and the total number of antibiotics, to which the isolates were exposed. MAR index values of >0.2 designate a high-risk source of contamination where antibiotics are often used [29].

### DNA extraction and hlyA gene detection through PCR

A single bacterial colony was cultured in nutrient broth (HiMedia) containing 2% NaCl (Ajax Finechem, Australia) and was incubated at 37°C overnight. The bacterial lysate was individually subjected to DNA extraction using the boiling method [30]. The hemolysis-associated gene determinants in the bacterial isolates were investigated by conventional PCR amplification of the *hly*A gene sequences using specific primers (F: 5′-GCCGAGCGCCCAGAAGGTGAGTT-3′ and R: 5′-GAGCGGCTGGATGCGGTTGT-3′) [31]. Each PCR reaction contained 12.5 μL of OnePCR™ master mix (GeneDireX, Taiwan), 0.2 μM of each forward and reverse primer (Thermo Fisher Scientific, USA), 2 μL of template DNA, and sterilized distilled water up to a total volume of 25 μL. Amplifications were performed using an Applied Biosystems™ Veriti™ 96-well thermal cycler (Thermo Fisher Scientific) under the following conditions: Initial denaturation at 95°C for 5 min for 1 cycle, 35 cycles consisting of denaturation at 95°C for 30 s, annealing at 55°C for 45 s and extension at 72°C for 1 min, and a final extension cycle at 72°C for 7 min. The obtained amplicons were detected by electrophoresis on a 1.5% (w/v) agarose gel at 100 V for 60 min and were visualized using the ChemiDoc MP imaging system (Bio-Rad, USA).

### Hemolysis phenotype and exoprotease assay

The hemolytic activity of the bacterial isolates secreting hemolytic enzymes that lyse red blood cells was investigated using blood agar as previously described [32]. All plates were incubated at 37°C for 24 h. The degree of hemolysis was visualized on blood agar plates. In addition, all isolates were screened for proteolytic activity using the skim milk plate method [33]. The plates were incubated at 37°C for 4 h. A clear zone around a bacterial colony indicated a positive result. The experiment was performed in triplicate.

### Quantitative biofilm formation assay in microtiter plates

The biofilm formation of bacterial strains was examined using the microtiter plate assay modified from the method previously mentioned [34]. In brief, bacteria were grown in Luria–Bertani broth (LB) containing 2% NaCl at 37°C for 24 h. Two hundred microliters of a 1:100 dilution of overnight cultures adjusted to 0.5 McFarland turbidity in fresh LB supplemented with 2% NaCl. Then, each diluted solution was dispensed into wells and plates were incubated at 37°C for 24 h. The control wells consisted of uninoculated LB with 2% NaCl. The contents of the wells were discarded and washed twice with phosphate buffer saline (PBS). Two hundred microliters of 1% (w/v) crystal violet dye were added to each well and the plates were subsequently incubated at room temperature (25°C) for 1 h. The staining dye was discarded, then the reaction wells were washed 3 times with PBS and were allowed to air-dry at 25°C. Two hundred microliters of 95% (v/v) ethyl alcohol were added to the dried wells and the plates were further incubated at 25°C for 5 min. Finally, the ethanol from each well was transferred to new microtiter plates and the optical density at 630 nm was measured. Three independent experiments were performed. The biofilm-producing capacity of all isolates was categorized as follows: No biofilm formation if OD_test_ < OD_control_; weak biofilm formation if OD_control_ < OD_test_ < 2OD_control_; moderate biofilm formation if 2OD_control_ < OD_test_ < 4OD_control_; and strong biofilm formation if OD_test_ > 4OD_control_ [7].

### Scanning electron microscopy (SEM)

The qualitative assay for biofilm formation was conducted using SEM, which was modified from a previously developed protocol [35]. After bacteria were allowed to form biofilms (at 12, 24, and 48 h), each reaction on a glass coverslip was rinsed 3 times with PBS solution. Then, the adhered cells and biofilms were fixed in 2.5% glutaraldehyde at 25°C for 1 h. After washing the reactions with 0.1 M sucrose phosphate buffer (SPB), then each well was fixed with 1% osmium tetroxide in SPB for 1 h. The fixed materials were treated with gradient ethanol (at 50%, 60%, 70%, 80%, and 90% and then twice at 100% for 15 min each) and were allowed to air-dry for 24 h. The dehydrated samples were coated with a 20 nm thick gold film using a sputter coater (Emitech K550, Ashford, UK) on an aluminum stub. Then, the biofilm formation was observed by SEM (JEOL JSM-6610LV, Japan). The electron microscope was operated at an accelerating voltage of 10 kV with a 13 mm working distance.

### Statistical analysis

All data were organized and analyzed using GraphPad Prism version 9 (La Jolla, CA, USA). The occurrence, antimicrobial resistance phenotype, hemolysis-associated genotype and phenotype, and biofilm formation phenotype of *Vibrio* and *Aeromonas* isolates were expressed as percentages.

## Results

### Occurrence of *Vibrio* and *Aeromonas* isolates in retail seafood samples

In a total of 120 retail seafood samples, 57 were found to be positive and, of these, 35 (61.4%) and 22 (38.6%) contained *Vibrio* and *Aeromonas* isolates, respectively. The isolated bacteria were from fish (19 isolates, 33.33%), shellfish (16 isolates, 28.07%), shrimp (11 isolates, 19.30%), squid (8 isolates, 14.04%), and crab (3 isolates, 5.26%). The isolates were further identified as *V. mimicus* (n = 7; 12.28%), *V. vulnificus* (n = 7; 12.28%), *A. hydrophila* (n = 6; 10.53%), *A. caviae* (n = 2; 3.51%), other *Vibrio* spp. (n = 21; 36.84%), and other *Aeromonas* spp. (n = 14; 24.56%). Table-1 shows the details of each isolate.

**Table 1:** Distribution of occurrence, antibiotic resistance profiles, and virulence of *Vibrio* spp. and *Aeromonas* spp. isolates.

Bacterial isolates	Source	Antibiotic - resistant profile	Bacterial strain	Presence of *hly* A gene	Hemolysis phenotype	Exoprotease production	Type of biofilm production
V1	Fish	-	*V. mimicus*	-	-	-	n
V2	Fish	AMC, AMP, SAM, and SXT	*A. hydrophila*	+	+	-	m
V3	Fish	-	*V. vulnificus*	-	-	-	n
V4	Fish	AMC, AMP, and SAM	*V. mimicus*	+	+	-	m
V5	Fish	-	*V. vulnificus*	+	+	-	m
V6	Fish	AMC, AMP, and SAM	*V. vulnificus*	+	+	-	n
V7	Fish	AMC and AMP	*Vibrio* spp.	+	+	-	n
V8	Fish	AMC, AMP, and SAM	*A. caviae*	+	-	-	s
V9	Fish	AMC, AMP, and SAM	*Aeromonas* spp.	+	+	-	m
V10	Fish	AMC, AMP, CAZ, SAM, and SXT	*Vibrio* spp.	+	+	-	m
V11	Fish	AMC, AMP, SAM, and TE	*Aeromonas* spp.	+	+	-	m
V12	Fish	AMC, AMP, SAM, SXT, and TE	*Aeromonas* spp.	+	+	+	m
V13	Fish	AMC, AMP, and SAM	*V. mimicus*	+	+	+	m
V14	Fish	AMC, AMP, and SAM	*Aeromonas* spp.	+	-	-	n
V15	Fish	SXT and TE	*Vibrio* spp.	+	+	-	m
V16	Fish	-	*Aeromonas* spp.	-	-	-	n
V17	Fish	AMC, AMP, SAM, and TE	*Vibrio* spp.	+	-	-	n
V18	Fish	AMC, AMP, and SAM	*Vibrio* spp.	+	+	-	n
V19	Fish	AMP and SAM	*Vibrio* spp.	+	+	+	s
V20	Shellfish	-	*V. vulnificus*	+	+	-	s
V21	Shellfish	-	*V. vulnificus*	+	+	-	n
V22	Shellfish	-	*V. mimicus*	+	+	-	m
V23	Shellfish	AMC	*Vibrio* spp.	+	+	-	s
V24	Shellfish	-	*Vibrio* spp.	-	-	-	m
V25	Shellfish	-	*Vibrio* spp.	+	+	-	s
V26	Shellfish	AMC, AMP, and SAM	*A. hydrophila*	+	+	-	m
V27	Shellfish	-	*Aeromonas* spp.	-	-	-	n
V28	Shellfish	AMC, AK, AMP, GN, and SAM	*Vibrio* spp.	+	-	-	n
V29	Shellfish	AMC, AK, AMP, CAZ, CTX, SAM, and SXT	*Aeromonas* spp.	+	-	-	m
V30	Shellfish	AMC, AK, AMP, CAZ, CTX, GN, SAM, and SXT	*Aeromonas* spp.	+	+	-	m
V31	Shellfish	AMC, AMP, and SAM	*Aeromonas* spp.	+	+	-	n
V32	Shellfish	-	*Vibrio* spp.	-	-	-	m
V33	Shellfish	-	*Vibrio* spp.	-	-	-	n
V34	Shellfish	-	*Vibrio* spp.	+	+	-	n
V35	Shellfish	-	*Vibrio* spp.	-	-	-	n
V36	Shrimp	AMC, AMP, and SAM	*A. hydrophila*	+	+	+	m
V37	Shrimp	AMC, AMP, SAM, and SXT	*A. hydrophila*	+	+	+	m
V38	Shrimp	AMC, AMP, and SAM	*A. hydrophila*	-	-	-	n
V39	Shrimp	-	*V. vulnificus*	+	+	-	m
V40	Shrimp	-	*V. vulnificus*	+	+	-	s
V41	Shrimp	AMC and AMP	*Vibrio* spp.	+	+	-	n
V42	Shrimp	AMC and AMP	*Aeromonas* spp.	+	-	-	n
V43	Shrimp	AMC, AMP, and SAM	*A. hydrophila*	+	+	-	s
V44	Shrimp	AMC, AMP, and SAM	*V. mimicus*	+	-	-	n
V45	Shrimp	AMP and SAM	*Aeromonas* spp.	+	+	-	s
V46	Shrimp	AMP, SAM, and SXT	*Aeromonas* spp.	+	+	-	m
V47	Squid	AMC, AMP, IMP, MEM, and SAM	*V. mimicus*	+	+	-	m
V48	Squid	TE	*V. mimicus*	-	-	-	n
V49	Squid	AMC, AMP, and SAM	*A. caviae*	+	+	-	n
V50	Squid	AMC, AMP, and SAM	*Aeromonas* spp.	+	+	-	m
V51	Squid	-	*Vibrio* spp.	-	-	-	n
V52	Squid	-	*Vibrio* spp.	+	+	-	s
V53	Squid	-	*Vibrio* spp.	-	-	-	m
V54	Squid	-	*Vibrio* spp.	-	-	-	m
V55	Crab	-	*Aeromonas* spp.	-	-	-	n
V56	Crab	-	*Vibrio* spp.	+	+	-	n
V57	Crab	-	*Vibrio* spp.	-	-	-	n
	Number of isolates (%)			43 (75.44)	36 (63.16)	5 (8.77)	32 (56.14)

AMC=Amoxicillin-clavulanate, AK=Amikacin, AMP=Ampicillin, CAZ=Ceftazidime, CTX=Cefotaxime, GN=Gentamicin; IMP=Imipenem, MEM=Meropenem, SAM=Ampicillin-sulbactam, SXT=Trimethoprim-sulfamethoxazole, TE=Tetracycline, +=Present, -=Not present; n=Non-biofilm producer, w=Weak biofilm producer, m=Moderate biofilm producer, s=Strong biofilm producer. *V. mimicus=Vibrio mimicus*, *V. vulnificus=Vibrio vulnificus*, *A. hydrophila=Aeromonas hydrophila*,

*A. caviae=Aeromonas caviae*

### Antimicrobial susceptibility profiles

Antibiotic sensitivity testing was performed on all isolated strains. In this study, resistance was observed against the following antimicrobial groups: Penicillin; AMP (n = 32; 56.14%), b-lactam combination agents; AMC (n = 30; 52.63%) and SAM (n = 29; 50.88%), cephalosporins; CTX (n = 2; 3.51%) and CAZ (n = 3; 5.26%), aminoglycosides; GN (n = 2; 3.51%) and AK (n = 3; 5.26%), carbapenems; MEM (n = 2; 3.51%) and IMP (n = 2; 3.51%), TEs; TE (n = 5; 8.77%) and folate pathways antagonisms; SXT (n = 8; 14.04%). The present study indicated that four types of antibiotics, namely, TZP, FEP, C, and CIP, are still effective against almost all tested isolates (Table-2). Moreover, the MAR index of the bacterial isolates obtained from seafood samples was calculated. Approximately 26 (45.61%) isolates were resistant to at least three classes of tested antibiotics with a MAR index of 0.2 or higher. By contrast, 23 isolates (40.35%) had a MAR index lower than 0.2. Nevertheless, eight (14.04%) isolates showed a MAR index of 0, indicating the susceptibility to all the antimicrobials tested. The most frequent MAR patterns were observed for AMC, AMP, and SAM, accounting for 15 (26.32%) isolates, followed by AMC and AMP, accounting for approximately 3 (5.26%) isolates, AMC and SAM; AMC, AMP, SAM, and TE; AMC, AMP, SAM, and SXT; and AMC, AMP, IMP, MEM, and SAM accounting for approximately 2 (3.51%) isolates. Interestingly, shellfish were the source of the greatest number of drug-resistant bacteria (AMC, CAZ, CTX, AMP, SAM, GN, AK, and SXT).

**Table 2:** Antibiogram phenotypes of bacterial isolates from seafood.

Antimicrobial agent (disk content)	Antimicrobial resistance patterns of bacterial isolates Number of isolates (%)		

	Susceptible	Intermediate	Resistant
Group penicillin			
Ampicillin (10 μg)	22 (38.60)	3 (5.26)	32 (56.14)
Group combined β-lactam agents			
Ampicillin-clavulanate (20/10 μg)	23 (40.35)	4 (7.02)	30 (52.63)
Ampicillin-sulbactam (10/10 μg)	28 (49.12)	0 (0)	29 (50.88)
Piperacillin-tazobactam (100/10 μg)	55 (96.49)	2 (3.51)	0 (0)
Group cephalosporin			
Cefepime (30 μg)	56 (98.25)	1 (1.75)	0 (0)
Cefotaxime (30 μg)	51 (89.47)	4 (7.02)	2 (3.51)
Ceftazidime (30 μg)	54 (94.74)	0 (0)	3 (5.26)
Group aminoglycoside			
Gentamicin (10 μg)	51 (89.47)	4 (7.02)	2 (3.51)
Amikacin (30 μg)	51 (89.47)	3 (5.26)	3 (5.26)
Group carbapenem			
Meropenem (10 μg)	55 (96.49)	0 (0)	2 (3.51)
Imipenem (10 μg)	55 (96.49)	0 (0)	2 (3.51)
Group chloramphenicol			
Chloramphenicol (30 μg)	51 (89.47)	6 (10.53)	0 (0)
Group tetracycline			
Tetracycline (30 μg)	48 (84.21)	4 (7.02)	5 (8.77)
Group fluoroquinolone			
Ciprofloxacin (5 μg)	52 (91.23)	5 (8.77)	0 (0)
Group folate pathway antagonist			
Trimethoprim/sulfamethoxazole (1.25/23.75 μg)	48 (84.21)	1 (1.75)	8 (14.04)

### Distribution of the *hlyA* gene, hemolysis phenotype, and proteolytic-producing strains

**Figure-1:**
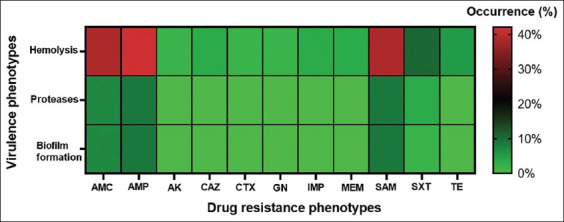
Heatmap of percent distribution for antimicrobial resistance phenotypes and virulence phenotypes of *Vibrio* isolates from seafood samples. The color band illustrates the percentages of virulence characteristics with individual drug-resistant phenotype. Generate using GraphPad Prism version 9 (La Jolla, CA, USA).

The distribution of the hemolysis-associated gene *hly*A was investigated for all *Vibrio* and *Aeromonas* isolates using PCR. The target PCR amplicons were 130 base pairs (bp) long. The data indicated that 43 (75.4%) isolates carried the *hlyA* gene and, among these, 36 (63.16%) showed hemolytic activity. In addition, the exoprotease-producing strain was detected in 5 (8.77%) isolates. A heatmap of the percent frequency of each virulence phenotype and antibiotic-resistant phenotype is shown in Figure-1.

### Biofilm formation capacity

**Figure-2:**
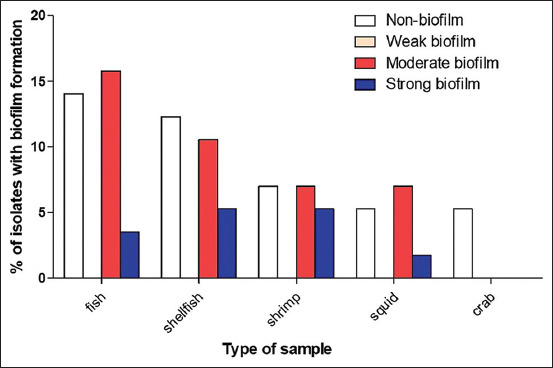
Biofilm formation potentials of bacterial isolated from different seafood samples using microtiter plate assay.

**Figure-3:**
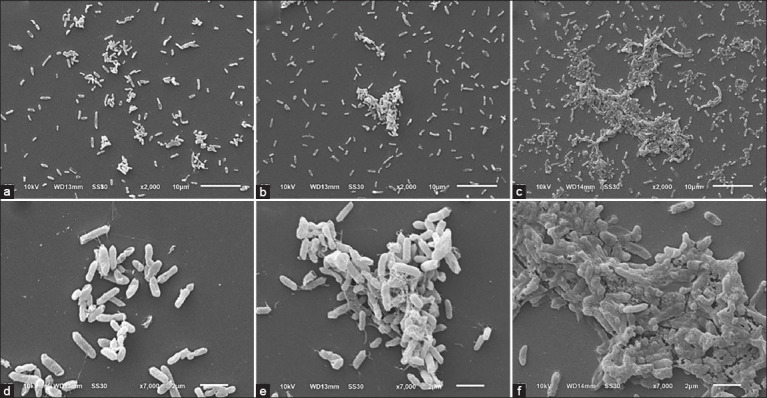
Representative scanning electron micrographs of biofilm formed by Vibrio isolate no. V19 at 37°C for 12 h (a, d), 24 h (b,e), and 48 h (c,f). Scale bars: 10 μm (upper panels) and 2 μm (lower panels).

The quantitation of biofilm formation for all isolates was determined using the microtiter plate assay. The results revealed the presence of approximately 32 (56.14%) biofilm-producing isolates that can be categorized into moderate biofilm producers, 23 (40.35%) isolates and strong biofilm producers, 9 (15.79%) isolates. No weak biofilm producers were found in this study (Figure-2). In addition, the ultrastructure of biofilm at different time points (12, 24, and 48 h) was revealed by SEM. With the increase in incubation time, more extensive biofilm could be observed, as shown in Figure-3.

## Discussion

At present, seafood is an important food source globally, including in Thailand. Seafood-related outbreaks are implicated in foodborne illnesses affecting more than half of a hundred thousand each year [36]. The contamination of seafood with pathogenic *Vibrio* and *Aeromonas* species is continuously reported [37–40]. In the past decades, in Thailand, these bacterial groups have been identified as the major causative agent of vibriosis [38, 41], which is caused by the consumption of raw seafood [6]. Items such as raw fish and shellfish can, actively or passively, contain pathogenic bacteria, which may be transmitted to humans. The results showed that most of the isolates belonged to the genus *Vibrio* and comprised mainly *V. mimicus* and *V. vulnificus*. Most isolates were found to contaminate fish products. This finding is in line with the results of the previous studies [38, 41, 42]. However, the bacterial species vary, possibly due to differences in the season and geographical origin, as well as in the reservoir host species. Furthermore, among *Aeromonas* spp., the predominantly isolated strain was *A. hydrophila* 6 (10.53%), which is commonly found in shrimp. This finding is in line with the results reported by Rahimi *et al*. [43].

Most bacterial strains were found to have reduced susceptibility to AMP 32 (56.14%), AMC 30 (52.63%), and ampicillin/sulbactam 29 (50.88%), but were still nonresistant to piperacillin/tazobactam, FEP, C, and CIP. Remarkably, shellfish were the source of the highest number of drug-resistant bacteria, which could specifically resist eight types of antibiotics tested, namely, AMC, CAZ, CTX, AMP, SAM, GN, AK, and SXT. This finding suggests that in the near future, commonly used antibiotics may become ineffective for the treatment of vibriosis [44]. In addition, most isolates 26 (45.61%) had extreme MAR index values of 0.2 and above, which is worrisome, as it indicates the presence of a greater incidence of multiple drug resistance (MDR) strains in the study area. This result is inconsistent with past research [45], which reported more than 80% of the isolates indicate high-risk origins of bacterial contamination. Nevertheless, the result indicates that fish are a high-risk source of contamination.

The presence of pathogenicity, as indicated by hemolytic and proteolytic activities, was also investigated. It was shown that most of the *hly*A-carrying strains 36 (63.16%) can cause red blood cells lysis and this result is in line with previously reported values showing that approximately 30%–100% of strains caused hemolysis [46]. Nevertheless, the present study detected a lower frequency of exoprotease-producing strains, indicating that the potential pathogenicity of seafood-derived bacteria is due to their possible public health risks. Furthermore, bacterial biofilm protects pathogens from environmental stress (such as antibiotics) and increases disease severity in the infected host [22–24]. In this study, the biofilm-producing abilities of the bacterial strains most resistant to multiple antibiotics were also investigated, providing significant evidence in the field.

The present study revealed the relationship between drug resistance patterns and virulence phenotype. The occurrence of antibiotic resistance was high for many types of antibiotics presently used. Most MDR isolates harbored the hemolysis-associated genes, *hly*A, and exhibited some virulence phenotypes and biofilm capacity. These results raise serious concerns regarding food safety and public health, as retail seafood products might serve as a reservoir for drug-resistant bacteria and virulent strains, which could be transmitted to humans through the food chain. Control strategies and microbiological risk assessments should be implemented. Similarly, constant monitoring of the occurrence, antimicrobial-resistant features, and virulence of foodborne organisms (particularly *Vibrio* spp. and *Aeromonas* spp.) can provide useful epidemiological information on foodborne bacterial infections in specific areas.

## Conclusion

This study examined the occurrence and phenotypic characterization of antibiotic resistance profiles and the distribution of biofilm formation among *Vibrio* and *Aeromonas* species isolated from seafood samples in Bangkok retail markets. It was clearly shown that the isolated bacteria presented multidrug resistance and some strains could produce biofilm. The results of this study may provide useful epidemiological information on foodborne bacterial infections in Thailand and also effective surveillance for infection control programs for vibriosis and *Aeromonas* transmission. However, there are limitations to the insight information about the drug resistance genes and AMR dynamics between commensals from gut microbiota and pathogens from the environment. Next-generation sequencing can address AMR encounters by enlightening novel perspectives on surveillance and risk appraisal. In addition, surveys of virulence phenotypes, antimicrobial resistance, and DNA fingerprints should be continuously conducted in the study area on a large scale.

## Authors’ Contributions

NI and SS: Conceived and designed the experiments. SS, WT, TK, and SA: Performed the experiments. PP and NS: Analyzed the data. NI: Contributed reagents, materials, and analysis tools. SS and NI: Wrote and edited the manuscript. All authors have read and approved of the final manuscript.

## References

[1] Austin B, Austin D.A, Munn C.B (2007). Bacterial Fish Pathogens:Disease of Farmed and Wild Fish Dordrecht.

[2] Tison D.L, Kelly M.T (1984). *Vibrio* species of medical importance. Diagn. Microbiol. Infect. Dis.

[3] Aarnisalo K, Raaska L, Wirtanen G (2007). Survival and growth of *Listeria monocytogenes* in lubricants used in the food industry. Food Control.

[4] Bintsis T (2017). Foodborne pathogens. AIMS Microbial.

[5] Dutta D, Kaushik A, Kumar D, Bag S (2021). Foodborne pathogenic vibrios:antimicrobial resistance. Front. Microbiol.

[6] Baker-Austin C, Oliver J.D, Alam M, Ali A, Waldor M.K, Qadri F, Martinez-Urtaza J (2018). *Vibrio* spp. infections. Nat. Rev. Dis. Primers.

[7] Maje M.D, Tchatchouang C.D.K, Manganyi M.C, Fri J, Ateba C.N (2020). Characterisation of *Vibrio* species from surface and drinking water sources and assessment of biocontrol potentials of their bacteriophages. Int. J. Microbiol.

[8] Qiao M, Ying G.G, Singer A.C, Zhu Y.G (2018). Review of antibiotic resistance in China and its environment. Environ. Int.

[9] Morris J.G., Acheson D (2003). Cholera and other types of vibriosis:A story of human pandemics and oysters on the half shell. Arch. Clin. Infect. Dis.

[10] Sack D.A, Sack R.B, Nair G.B, Siddique A.K (2004). Cholera. Lancet.

[11] *Vibrio* Species Causing Vibriosis (2022). *Vibrio* and Food.

[12] Ali M, Lopez A.L, You Y, Kim Y.E, Sah B, Maskery B, Clemens J (2012). The global burden of cholera. Bull. World Health Organ.

[13] Vila J, Pal T (2010). Update on antibacterial resistance in low-income countries:Factors favoring the emergence of resistance. Open Infect. Dis. J.

[14] Raszl S.M, Froelich B.A, Vieira C.R.W, Blackwood A.D, Noble R.T (2016). *Vibrio parahaemolyticus* and *Vibrio vulnificus* in South America:Water, seafood and human infections. J. Appl. Microbiol.

[15] Figueras M.J (2005). Clinical relevance of *Aeromonas* sM503. Rev. Med. Microbiol.

[16] Illanchezian S, Jayaraman S, Manoharan M.S, Valsalam S (2010). Virulence and cytotoxicity of seafood borne *Aeromonas hydrophila*. Braz. J. Microbiol.

[17] Horseman M.A, Surani S (2011). A comprehensive review of *Vibrio vulnificus*:An important cause of severe sepsis and skin and soft-tissue infection. Int. J. Infect. Dis.

[18] Igbinosa I.H, Igumbor E.U, Aghdasi F, Tom M, Okoh A.I (2012). Emerging *Aeromonas* species infections and their significance in public health. ScientificWorldJournal.

[19] Lopatek M, Wieczorek K, Osek J (2018). Antimicrobial resistance, virulence factors, and genetic profiles of *Vibrio parahaemolyticus* from seafood. Appl. Environ. Microbiol.

[20] Rasmussen-Ivey C.R, Figueras M.J, McGarey D, Liles M.R (2016). Virulence factors of *Aeromonas hydrophila*:In the wake of reclassification. Front. Microbiol.

[21] Mizuno T, Debnath A, Miyoshi S.I (2019). Hemolysin of *Vibrio* species. In:Microorganisms.

[22] Flemming H.C, Wingender J (2010). The biofilm matrix. Nat. Rev. Microbiol.

[23] Mizan M.F.R, Jahid I.K, Ha S.D (2015). Microbial biofilms in seafood:A food-hygiene challenge. Food Microbiol.

[24] Simões M, Cleto S, Pereira M.O, Vieira M.J (2007). Influence of biofilm composition on the resistance to detachment. Water Sci. Technol.

[25] Elexson N, Afsah-Hejri L, Rukayadi Y, Soopna P, Lee H.Y, Zainazor T.T, Son R (2014). Effect of detergents as antibacterial agents on biofilm of antibiotics-resistant *Vibrio parahaemolyticus* isolates. Food Control.

[26] International Organization for Standardization. ISO 21872-1:2017:Microbiology of the Food Chain--Horizontal Method for the Determination of *Vibrio* spp. Part 1, Detection of Potentially Enteropathogenic *Vibrio parahaemolyticus *Vibrio cholerae* and *Vibrio vulnificus*, 2017* (2017). International Organization for Standardization, Geneva, Switzerland.

[27] Baur A.W, Kirby W.M.M, Sherris J.C.T, Turch M (1966). Antibiotic susceptibility testing by a standardized single disk method. Am. J. Clin. Pathol.

[28] Clinical and Laboratory Standards Institute. Performance Standards for Antimicrobial Susceptibility Testing (2011). Twenty-First Informational Supplement M100-S21.

[29] Krumperman P.H (1983). Multiple antibiotic resistance indexing of *Escherichia coli* to identify high-risk sources of fecal contamination of foods. Appl. Environ. Microbiol.

[30] Dashti A.A, Jadaon M.M, Abdulsamad A.M, Dashti H.M (2009). Heat treatment of bacteria:A simple method of DNA extraction for molecular techniques. Kuwait Med. J.

[31] Wang G, Clark C.G, Liu C, Pucknell C, Munro C.K, Kruk T.M, Rodgers F.G (2003). Detection and characterization of the hemolysin genes in *Aeromonas hydrophila* and *Aeromonas sobria* by multiplex PCR. J. Clin. Microbiol.

[32] Brumfield K.D, Carignan B.M, Ray J.N, Jumpre P.E, Son M.S (2017). Laboratory techniques used to maintain and differentiate biotypes of *Vibrio cholerae* clinical and environmental isolates. J. Vis. Exp.

[33] Filloux A, Ramos J.L (2014). Pseudomonas Methods and Protocols. Humana Press, Totowa, New Jersey.

[34] O'Toole G.A, Pratt L.A, Watnick P.I, Newman D.K, Weaver V.B, Kolter R (1999). Genetic approaches to study of biofilms. Meth. Enzymol.

[35] Rodrigues S, Paillard C, Le Pennec G, Dufour A, Bazire A (2015). *Vibrio tapetis*, the causative agent of brown ring disease, forms biofilms with spherical components. Front. Microbiol.

[36] Vi Butt A.A, Aldridge K.E, Sanders C.V (2004). Infections related to the ingestion of seafood Part I:Viral and bacterial infections. Lancet Infect. Dis.

[37] Elhadi N, Radu S, Chen C.H, Nishibuchi M (2004). Prevalence of potentially pathogenic *Vibrio* species in the seafood marketed in Malaysia. J. Food Prot.

[38] Woodring J, Srijan A, Puripunyakom P, Oransathid W, Wongstitwilairoong B, Mason C (2012). Prevalence and antimicrobial susceptibilities of *Vibrio*, *Salmonella*, and *Aeromonas* isolates from various uncooked seafoods in Thailand. J. Food Prot.

[39] Sathiyamurthy K, Baskaran A, Subbaraj D.K (2013). Prevalence of *Vibrio cholerae* and other vibrios from environmental and seafood sources, Tamil Nadu, India. Br. Microbiol. Res. J.

[40] Song X, Zang J, Yu W, Shi X, Wu Y (2020). Occurrence and identification of pathogenic *Vibrio* contaminants in common seafood available in a Chinese traditional market in Qingdao, Shandong Province. Front. Microbiol.

[41] Yano Y, Hamano K, Satomi M, Tsutsui I, Ban M, Aue-Umneoy D (2014). Prevalence and antimicrobial susceptibility of *Vibrio* species related to food safety isolated from shrimp cultured at inland ponds in Thailand. Food Control.

[42] Raissy M, Khamesipour F, Rahimi E, Khodadoostan A (2014). Occurrence of *Vibrio* spp., *Aeromonas*
*hydrophila*, *Escherichia coli* and *Campylobacter* spp. in crayfish (*Astacus leptodactylus*) from Iran. Iran. J. Fish. Sci.

[43] Rahimi E, Raissy M, Razzaghimanesh M, Dastgerdi A.A, Shahraki M.M (2014). Occurrence of *Aeromonas hydrophila* in fish, shrimp, lobster and crab in Iran. Kafkas Univ. Vet. Fak. Derg.

[44] Shaw K.S, Goldstein R.E.R, He X, Jacobs J.M, Crump B.C, Sapkota A.R (2014). Antimicrobial susceptibility of *Vibrio vulnificus* and *Vibrio parahaemolyticus* recovered from recreational and commercial areas of Chesapeake Bay and Maryland Coastal Bays. PLoS One.

[45] Letchumanan V, Pusparajah P, Tan L.T.H, Yin W.F, Lee L.H, Chan K.G (2015). Occurrence and antibiotic resistance of *Vibrio parahaemolyticus* from shellfish in Selangor, Malaysia. Front. Microbiol.

[46] Bhowmik P, Bag P.K, Hajra T.K, De R, Sarkar P, Ramamurthy T (2009). Pathogenic potential of *Aeromonas hydrophila* isolated from surface waters in Kolkata, India. J. Med. Microbiol.

